# On the potential genotoxic/mutagenic effects of peroxide-treatment on organic fractions of thermal–mineral waters

**DOI:** 10.1007/s00484-026-03304-3

**Published:** 2026-09-07

**Authors:** Csaba Varga

**Affiliations:** Department of Public Health Medicine, Medical School, University of Pécs, Pécs, Hungary

## Introduction

The thermal-medicinal waters of the Carpathian (Pannonian) Basin contain high concentration of organic compounds. (Varsányi et al. 2002; Fekete et al. 2009, 2012) Consequently, the chemical disinfection of spa pools which is mandated by the health authorities in some countries (Pobat et al. 2026) can cause serious problems from a public health perspectives. The microbiological safety of thermal waters improves as a result of disinfection alone; however, the formation of by-products could result in a chance of exposure to toxic chemicals for the balneotherapy patients and healthy spa users. It has been known for decades from research into drinking water, during chlorination, numerous mutagenic/carcinogenic by-products are formed from organic traces (IARC 1991, 1997). In the case of organic-rich spa waters this potential could be extraordinary, decreasing safety and perhaps medical efficiency of bathing and other balneotherapeutic treatments (Varga 2012, 2019).

Our aim was to study how organic fractions of spa waters react to hydrogen-peroxide treatment, which is recommended, for example, by the Hungarian Public Health Authority, *nota bene* without any prior relevant modelling having been carried out on the formation of by-products.

Our original question was whether genotoxicity (mutagenicity) could be detected in treated organic content of the medicinal waters. To investigate mutagenic effects the Salmonella/Ames mutagenicity assay was used. This study was a model study as we treated the organic fractions of the water samples directly, rather than the waters themselves. The organic content was concentrated as it is necessary for bacterial mutagenicity studies. As we did not study the original spa water but its preparation, all possible biological/microbial effects were also avoided. These and further factors should be considered in a more complex study.

In the study conducted the Salmonella TA 100 strain was sensitive to the base pair substitution, whilst TA 98 was sensitive to frame-shift mutations. Metabolic activation indicated the presence of indirect mutagens that require enzymatic activation and produce mutagenic metabolites. Four different organic preparations derived from thermal-medicinal waters water organic preparations were tested before and after peroxidation with a proposed concentration.

## Materials and methods

### Sample preparation

A patented method was employed to prepare 3000-fold organic isolates from four thermal-medicinal water wells (Varga et al. 1992). These ethanolic-aqueous isolates were used in the *Salmonella/microsome Ames mutagenicity test*. The isolates represented the thermal waters of Debrecen (De), Hajdúszoboszló (Hs), Szigetvár (Sg) and Kakasszék (Ks), respectively. (The *Dr.Balneo*^®^ commercial preparations were produced using the mobile equipment of Aquakoncentrátum Ltd., Budapest, Hungary). The 2 mL samples were treated with H_2_O_2_ (Molar, Budapest) at a concentration equivalent to 50 mg/L. (“Hyperol” carbamide-peroxide tablets produce hydrogen-peroxide solution in aqueous medium.) Samples were incubated overnight at 37 °C in a rotary incubator then filtered through a syringe fitted with 0.45 μm membrane filter disc (Nalgene, USA). The contact time represented the filling cycles of the basin (10–12 h). Peroxide concentration applied in our study based on the proposed final concentration of the disinfectant producers and empirical studies. 0 mg/L peroxide could be measured by test strips (Vansful, 0–2 mg/L, accuracy: 0.1) at the end of the contact time.

The “untreated” controls were prepared in the same way, omitting only the peroxide-treatment.

### Ames test

*S. Typhimurium* TA98 and 100 strains were applied, in the pour-plate method with and without metabolic activation (+/–S9). The untreated and peroxidised samples were tested at concentrations of 0 (solvent control), 25, 75, and 100 µL, respectively, on three parallel plates, alongside strain-specific positive controls, in accordance with the standard procedure. (Maron and Ames 1983). The tests were repeated twice. The calculated means always represented 6 plates (number of revertant colony).

### Statistical analysis

The results were evaluated as “positive” strain-specific response (++), if the number of revertants doubled (TA100) or tripled (TA98) compared with the corresponding control (Levy et al. 2019). In addition, paired “t-tests” were also performed and differences at a significance level of *p* < 0.05 were also marked with a (+) symbol.

## Results

The 100 µL doses proved to be toxic in our test system; therefore, we used plates containing 75 µL of samples (highest evaluable doses) as controls. The 75 µL dose of peroxide-treated samples were compared with the untreated paired control. (The 25 µL doses of peroxidised samples were also tested.) The control plates contained the highest non-toxic dose (75 µL) of the original, untreated preparation. If, for at least one dose among peroxide-treated samples the mutation frequency doubled or tripled (++) or increased significantly (+) compared with the untreated control, then the test was considered as positive. Summarized data are presented in Table 1 (See details in the Supplementary File). 

**Table 1:** Mutagenicity pattern of organic isolates in the Ames mutagenicity assay. Applied doses: 75μL/plate. Chemical Oxygen Demands (COD) measured with dichromate method are also indicated. (++: doubling of revertant numbers, +: significance at p<0.05)

Sample (COD_dichr_ mg/L)	TA 98 -S9	TA 98 +S9	TA 100 -S9	TA 100 +S9
De (37)	–	–	–	–
Hs (42)	–	–	–	–
Ks (48)	–	+	++	+
Sg (38)	–	+	–	–

## Discussion

As public health authorities in Central and Eastern European countries view the microbiological quality of bathing waters with concern, they are showing great interest in the disinfection of such waters. At the same time, therapeutic water is not simply swimming pool or recreational water. Any changes to its therapeutic effects must also be taken into account. For this reason alone, it is important to examine the therapeutic efficacy of disinfected water, as well as the formation of by-products potentially harmful to health.

The authors from the Hungarian Public Health Authority believe that H_2_O_2_ does not reduce the therapeutic effect, as of the three presumed active ingredients, only the sulphide ion concentration decreased (–91%) (Gere et al. 2022a, b). However, the model used was not suitable for investigating the actual situation. It is a serious mistake to assume that the presence of a few inorganic components could explain the therapeutic effect. There is wealth of direct and indirect evidence to the contrary (Varga 2010; Hanzel et al. 2019). Although sulphide, iodide, and bromide ions may be absorbed in the intestines during the consumption medicinal water, there are no quantitative data on their dermal absorption. Furthermore, in many cases the therapeutic effect is attributable to the organic fraction rather than to the inorganic components. (Among inorganic compounds, important to note, it is precisely sulphide that may possess therapeutic potential (Fioravanti et al. 2011; Cheleschi et al. 2020). Another (perhaps the greatest) problem with their claim is that residual therapeutic effects of the disinfected thermal-mineral water must be demonstrated by a randomized controlled clinical trial. (Tefner et al. 2012; Hanzel et al. 2019)

There are no data in the literature on the treatment of drinking water, swimming pool water or therapeutic water with H_2_O_2_. Literature focuses the combined UV/H_2_O_2_ or chlorine+activated carbon+peroxide treatment highlighting the formation of mutagenic by-product formation (Metz et al. 2011; Hofman-Caris et al. 2015; Totaro et al. 2019), or ecological effects of peroxide on natural fresh waters (Almeida et al. 2026).

In the present study, mutagenicity was detected in two samples following the proposed H_2_O_2_ disinfection. Unsurprisingly, the sample prepared from the highest COD water (richest in organics) exhibited the most significant effect (K**s**). Its mutagenic spectrum was broad (3 out of 4 conditions). Another sample (Sg) also showed but weak mutagenicity (1 condition).

It is important to emphasize that residual peroxide could not cause mutagenicity detected as peroxide completely decayed until the end of the incubation process.

In conclusion, chemical disinfection of some types of thermal-mineral (organic-rich) waters can generate genotoxic by-products that could even lead to serious health damage, since, for example, carcinogens have stochastic effects without any threshold. If this is true, then peroxidation cannot be an effective method for protecting the health of spa water users, even if the microbiological quality of the water improves. (Varga 2019)

In the author’s opinion, therapeutic waters should not be chemically treated until their therapeutic mechanism of action is known. This is because such treatment could not only compromise the bathing experience and therapeutic effects, but could also lead to hazardous exposure. This could diminish the tourist appeal of therapeutic waters and may even be harmful to health.

## Supplementary Information

Below is the link to the electronic supplementary material.

Supplementary Material 1

## Data Availability

All data included.
