# Supplementary material for: On the potential genotoxic/mutagenic effects of peroxide-treatment on organic fractions of thermal–mineral waters
Source: Int J Biometeorol. 2026 Sep 7;70(9):249. doi: 10.1007/s00484-026-03304-3 (PMC13550015; doi:10.1007/s00484-026-03304-3)
Supplement: Supplementary file 1 [file 484_2026_3304_MOESM1_ESM.docx]

| **TA98**  **–S9** | **revertant/plate** | **µL/plate** | **TA100 –S9** | **revertant/plate** | **µL/plate** |
| --- | --- | --- | --- | --- | --- |
|  |  |  |  |  |  |
| blind | 21.4±4.7 | 75 | blind | 95.5±18.0 | 75 |
|  |  |  |  |  |  |
| untreated: |  |  |  |  |  |
| Db | 24.3±6.7 | 75 | Db | 119±15.2 | 75 |
| Hs | 31.0±4.9 | 75 | Hs | 118.3±15.0 | 75 |
| Sg | 35.3±5.0 | 75 | Sg | 108.0±3.6 | 75 |
| Ks | 38.6±8.1 | 75 | Ks | 127.8±36.5 | 75 |
|  |  |  |  |  |  |
| H_2_O_2_-treated: |  |  |  |  |  |
| Db | 29.3±6.8 | 75 | Db | 135±12.7 | 75 |
| Hs | 39±9.8 | 75 | Hs | 135±17.8 | 75 |
| Sg | 33±8.0 | 75 | Sg | 139.3±6.5 | 75 |
| Ks | 37.5±0.7 | 75 | Ks | 277±43.5*** | 75 |
| Db | 30.6±3.5 | 25 | Db | 114.6±11.0 | 25 |
| Hs | 28.6±7.0 | 25 | Hs | 127.6±9.6 | 25 |
| Sg | 30.6±3.7 | 25 | Sg | 117.6±5.6 | 25 |
| Ks | 32.6±14.2 | 25 | Ks | 207±4.0* | 25 |

*significant at p<0.05; ***doubling of revertant number (as compared to the untreated sample)

| **TA98 +S9** | **revertant/plate** | **µL/plate** | **TA100 +S9** | **revertant/plate** | **µL/plate** |
| --- | --- | --- | --- | --- | --- |
|  |  |  |  |  |  |
| blind | 25.0±3 | 75 | blind | 98.6±27.3 | 75 |
|  |  |  |  |  |  |
| untreated: |  |  |  |  |  |
| Db | 24±2.4 | 75 | Db | 117.5±13.4 | 75 |
| Hs | 25±5.5 | 75 | Hs | 96±15.5 | 75 |
| Sg | 34.3±7.5 | 75 | Sg | 124±0.0 | 75 |
| Ks | 35±9.1 | 75 | Ks | 102±15.5 | 75 |
|  |  |  |  |  |  |
| H_2_O_2_-treated: |  |  |  |  |  |
| Db | 31.8±7.1 | 75 | Db | 127±45.2 | 75 |
| Hs | 30.3±1.5 | 75 | Hs | 131±9.8* | 75 |
| Sg | 24.5±17.6 | 75 | Sg | 132.6±7.1 | 75 |
| Ks | 61.6±7.7* | 75 | Ks | 129±19.0 | 75 |
| Db | 31.6±7.3 | 25 | Db | 100.7±7.3 | 25 |
| Hs | 24.6±10.4 | 25 | Hs | 129±31.1 | 25 |
| Sg | 58.4±9.5* | 25 | Sg | 108.6±8.5 | 25 |
| Ks | 48.6±13.8 | 25 | Ks | 132.5±6.3* | 25 |

*significant at p<0.05; (as compared to the untreated sample)
